# The Science of Medicine and the Healing Art1Read at the thirty-fourth annual meeting of the Medical Association of Central New York, held at Buffalo, N. Y., August 27 and 28, 1901.

**Published:** 1901-11

**Authors:** John L. Heffron

**Affiliations:** Syracuse, N. Y., Professor of clinical medicine, Syracuse University


// BUFFALO MEDICAL JOURNAL.

VOL. XLI.—LVII. NOVEMBER, 1901. NO. 4.

### ORIGINAL COMMUNICATIONS.

### The Science of Medicine and the Healing Art.[1]

By JOHN L. HEFFRON, M. D. Syracuse, N. Y.,
Professor of clinical medicine, Syracuse University.

THE various reviews of the progress of the science and art of medicine during the century just closed are worthy of careful study by all intelligent men. From them I propose to make certain deductions concerning the present status of medicine, and upon them base some conclusions affecting medical legislation for the twentieth century.

The history of the evolution of the science of medicine should be well understood and kept constantly in mind if one would clearly understand the difficulties with which the practice of medicine is still beset. The influence in medical practice of the priestly method, which survives today, is of itself, and without historical data, sufficient proof of the origin of the healing art. From mystery through empiricism to the science of the present, medicine has passed, making a history of unequaled interest.

The science of medicine is founded on a complete knowledge of the structure and the functions of the body as a whole and of each component organ and tissue, of the way by which it is reproduced and the method of its development. The sciences of anatomy, histology, physiology, organic and physiological chemistry and embryology, while essentials to the physician, are the common property of all educated people. They constitute a large part of the recognised courses in sciences in all our uni-

1. Read at the thirty-fourth annual meeting of the Medical Association of Central New York, held at Buffalo, N. Y., August 27 and 28, 1901.

versities and concerning their status there is no question, not even in the minds of the most prejudiced dogmatist.

That the past has developed a science of pathology is capable of demonstration to any seeker after the truth. That frost and fire and the poisons of animals, insects and plants produce certain and constant effects upon exposed parts is a part of the experimental knowledge of mankind. No less certain, no less constant and no less invariable effects upon organs and tissues are produced by the various diseases which affect the body, and no less clear is the demonstration, though it requires the scalpel and microscope to reveal it and the educated eye to appreciate it. Pathology is a science.

Every man that is born of woman has experienced disease. The search for its causes has been never ceasing. Demonology, mysticism, superficial observation and philosophic speculation dominated this field up to the middle of the nineteenth century. Then, with much opposition from theologians and philosophers, there was evolved the scientific method of experimentation and demonstration, by which every ancient proposition was tried and its truth or falsity established by methods which are now so universally recognised, that to this generation it seems impossible that they ever could have encountered opposition. By this method there have been established, with little chance of question, the absolute determining causes of most of the recognised diseases, while the study of man and his environment has thrown clear light upon the causes which predispose to disease. The most brilliant demonstrations in the last quarter of the nineteenth century and the most far-reaching in their results, were those of Lister, Pasteur and Koch and their co-laborers. Upon their demonstrated results there has been established the science of etiology.

The natural history of diseases, by which I mean a knowledge of their inception, their development, their natural tendencies and terminations, as manifested by symptoms and physical and vital phenomena, is scientific knowledge. The collected and corrected observations of astute clinicians of the past give us a most accurate picture of each disease that is known, so that the recognition of them is possible. The practical application of such knowledge by one technically trained to the accurate use of all the organs of special sense and to the use of all the instruments of precision and to the laboratory method, constitutes the art of diagnosis. It would be as difficult to exaggerate a statement of the advancement of the methods of diagnosis which have been made during the last quarter of the nineteenth century as to magnify their importance. Among scientific physicians accurate diagnosis is recognised as of primary importance. Indeed, much of the opposition to modern medicine is due to the fact that it has seemingly been considered by a large school of medical men to be not simply the flower of medical education but to be actually the fruit. If such have not taught they have caused many to feel that when an unquestionable diagnosis has been established, any further active interest in the case dies naturally to be awakened only by a post-mortem examination. While much of the reactionary tendencies in medical practice in recent years has been fostered, if not caused by this attitude of some of the leaders in medical thought, it is a pleasure to record that the reviews of the last year indicate that at the dawn of the twentieth century, diagnosis is considered the necessary preliminary step to a scientific therapeusis.

Up to this point it is clear from a history of the past that medicine may now rightfully be considered a science, and upon this deduction all who are at present recognised as physicians by the State of New York are agreed. All recognise as sciences and as essential to the medical practitioner not only the fundamental sciences but pathology, etiology, including bacteriology, the natural history of disease and diagnosis.

I believe that the history of the past also teaches that there is a science of therapeutics. The scientific method of investigation has taught us first the natural tendencies of disease and how they are modified by heredity and environment. It has demonstrated the natural conditions under which diseases are prevented and are overcome. It has demonstrated by experimentation on animals and by observation on man, how various remedial agents modify the course of disease. While it is true that we have as yet but few specific remedies, it is also true that the experimental method has demonstrated the exact physiological action of most of the remedies at present used by the educated physician. To ignore such facts seems to us inexcusable, and to make use of such facts, while professing a belief in a therapeutic dogma incapable of proof by the scientific method, seems dishonest. But in practical therapeutics, many things have to be reckoned with besides our knowledge of disease, of its natural curative tendency and of the action of remedial agents. The ignorance, prejudices, emotions and egotism of the sick make them easily dupes and the world has never lacked those who prey upon them.

Huxley said in a humorous letter to his daughter: "Men, my dear, are very queer animals; a mixture of horse-nervousness, ass-stubbornness and camel-malice, with an angel bobbing about unexpectedly like the apple in the posset and when they can do exactly as they please, they are very hard to drive." People who are so divided as to the proper method of saving their souls that religious sects multiply constantly, can hardly be expected in this century to be a unit on the proper method of saving their bodies. It will be difficult if not impossible for scientific physicians to persuade the people that they are actuated by purely philanthropic motives in pressing legislation upon them which may make their choice of methods of healing impossible or limited. Our purpose may be never so pure, our zeal know no langor, our reasoning logical and flawless, our demonstration of blessings already conferred on the people by scientific control of the great plagues, like smallpox, enforced by legislative enactment, most convincing and yet over and over again you will have the spectacle of delightful men like Mark Twain and Will Carleton, standing as champions for the right of people to a free choice of the method by which they shall be cured or killed. It is the easiest thing in the world to establish a doctrine and freedom to believe in it, however erroneous or even harmful it may be, is looked upon as an inalienable right. The profession long ago recognised the fallibility of statistics and knows that any number of facts can be arrayed in support of any proposition however absurd. But in the minds of the people, there is no distinction between post hoc and propter hoc, and testimony of benefit received after any treatment is convincing, even to a mind educated in all but the special field in which we labor. However much historical criticism may negative miracle, yet echoing down the centuries, convincing to the people, comes such expressions as this: "One thing I know; whereas I was blind now I see."

None can deprecate sectarianism more than I. It has served only to obscure the truth in every department of human knowledge. The zeal with which any particular dogma is accepted tends but to blind the intellect to a perception of its true importance and its relationship. It is so in theology, in politics, in medicine. But so long as human nature is as it is, and is fallible, so long will dogma gain possession of the minds of the multitude. In matters of belief and of what one considers to be of belief, there will be no possibility of proving in the twentieth century that only one belief is right. Such considerations, it seems to me, must have been the foundation of the recognition by the legislature of our state of the medical sects known as homeopathy and eclecticism. When this state legally recognised the physicians at that time practising within her borders and classified them in three groups, regular, homeopathic and eclectic, she at the same time made our present law requiring all candidates for the profession of medicine to pass examinations identical in all branches save in those pertaining to the practice of medicine. I think all older physicians who have lived through this discussion will agree that the results to the people have justified the law. Without any question whatever the making of the law has greatly improved the quality of the average medical practitioner in our state. A uniform standard for all save in therapeutics has compelled a higher and more thorough education of all. A study of the curricula of the various medical schools, more particularly of those in which therapeutic dogma is taught, is proof sufficient of the truthfulness of this proposition. I am connected with a medical school that has always commanded respect for its high standards, voluntarily set up when higher medical education was the exception in our nation, but I can testify that this law has been so well enforced, that the state examinations by the licensing board appear to the best of our students as a thorough test of scholarship. This is also true in those coming from sectarian schools. The result is that the state has a body of medical practitioners, grown up since the establishment of this law, of more than ordinary qualifications. The beneficent results have been felt by the people whether they know it or not. The grounding of all in the science of medicine has greatly modified the peculiar dogmas of practice and has been a process of enlightenment that has done more toward minimizing the evils of what we believe to be false doctrine than centuries of direct opposition.

But these examinations present some anomalies which we should correct and I believe the times are now ripe for considering them. I know not by whose authority the single individualised examination for each special group of physicians was entitled, "Therapeutics, practice and materia medica," but I believe I know that all physicians recognised by the state will agree that there is no dogma in the knowledge of the natural history of disease which must be what is meant by "practice" independent of materia medica and therapeutics. In each of the papers prepared by the committee, selected by the New York State Medical Society for the past few years, there is one or more questions requiring a description of the natural history of a disease with no inquiry as to the treatment, and which every candidate for a license to practise should answer. In our efforts to improve medical legislation, let us correct this anomaly first, and agree to request the regents to make the individualised examination one in "materia medica and therapeutics" only.

On the other hand, there is no title under which there can be required a description of the natural history of disease and this is a grave omission. Inasmuch as our continental confrères have for many years used the term, "internal medicine" as a convenient title for this subject, I would suggest that this term be adopted in the state examinations, as it is already well known in medical literature.

If it is not advisable to increase the number of subjects for examination, it would seem to me in harmony with modern tendencies in medical education to relegate "chemistry," by which is generally understood inorganic chemistry to the entrance examinations. In that case physiological chemistry would be understood to belong to physiology and organic chemistry to both physiology and pathology and would be included in the final examination in those two subjects. Pathology, being related to both surgery and internal medicine should be a subject for examination by itself. We would then have the following subjects for the examinations for a license to practise medicine in our state, viz.: anatomy, physiology and hygiene, pathology, internal medicine and diagnosis, surgery, obstetrics, materia medica and therapeutics. This would be harmonising our examinations with the spirit of the law and the letter could be made to conform by slight amendments to the existing law. A minute to this effect forwarded from this body to the committee on legislation of the New York State Medical Society would meet with respectful consideration, and if I do not greatly mistake the spirit of physicians not in affiliation with us, a similar minute to their proper committee would be respectfully received. It seems to me that this is the first essential step in considering other medical legislative acts which must of a certainty come up this winter. It is absolutely impossible to unify medical practice.

There are no two physicians in any single school whose practice and methods are identical, however similar they may be. It is a curious phenomenon that the majority of those who have emancipated themselves from theological dogma have embraced medical dogma because it is a late efflorescence and because the new is supposed to be the advanced thought. As before said the multitude claim the right to believe what appeals to their reason and no body of men can prevent it. On the other hand, I am convinced that the workings of the present medical law have been so favorable, that it will not be difficult to persuade the people and their representatives in legislative halls of the truth of the fundamental principle, that he who would practice the healing art by any method whatsoever should be compelled to show proof of his knowledge of the human body and of the diseases which it suffers, and should be able to tell what is the matter with a sick man before he applies his remedy. If we use the term "one who practises the healing art" instead of "one who practises medicine" which to most seems to indicate that such an one must use drugs, it will be seen instantly that it of necessity includes all of the latter day candidates for financial support by sick people whether they be termed "mental healers," "faith curers," "christian scientists," "osteopaths," or what nots. If we could secure an amendment to the present medical law, whereby all those who propose to practise the healing art must pass examinations identical in all subjects save in materia medica and therapeutics or the application of remedial measures and leave such a person as free in the exercise of his art as we ourselves are from our very lack of limitations, there can be no question but that we would serve the people, and we would be benefactors to the very ones whose errors we deplore.

528 S Salina Street.

